# Litigation analysis of medical damage after total knee arthroplasty: a case study based on Chinese legal database in the past ten years

**DOI:** 10.1186/s42836-022-00141-8

**Published:** 2022-10-01

**Authors:** Shuai Liu, Jilong Zou, Shuai Wang, Guangyu Liu, Yan Zhang, Shuo Geng

**Affiliations:** Department of Orthopedics, the First Affiliated Hospital of Harbin Medical University, Harbin, 150001 China

**Keywords:** Total knee arthroplasty, Litigation, Medical damage

## Abstract

**Background:**

The medical damage litigations after knee arthroplasty are on the rise year by year. However, few studies examined the litigation after knee arthroplasty. This study analyzed the litigation of medical damage after knee replacement in the past ten years based on a Chinese database. It synthesized the focus of the dispute in these cases to provide a reference for doctors to reduce the risk of litigation.

**Methods:**

Retrospectively analyzed were medical damage litigations after total knee arthroplasty in the past ten years (June 2011–June 2021) from the "Wolters Kluwer Legal Information Database". The data collected included the characteristics of patients, causes of litigation, the results of litigation and the amount of compensation.

**Results:**

A total of 110 litigation cases were analyzed, including 40 male patients (36.3%) and 70 female patients (63.6%). The top cause of litigation was infection (43.6%). The most common factor leading to the doctor losing the case was "complications caused by operational error" (*P* < 0.05). Complications, such as amputation, postoperative ischemic stroke and infection, were more likely to result in higher compensation.

**Conclusions:**

The prevention of infection and the avoidance of operational errors are very important in avoiding medical litigations. Moreover, avoiding disabling complications or a protracted course of disease could significantly reduce the amount of compensation. In addition, full and reasonable communication, paying full attention to the reaction of patients, and timely diagnosis could also effectively minimize the risk of litigation and loss.

## Background

Total knee arthroplasty (TKA) has been successfully used to treat chronic degenerative diseases of the knee joint for over 50 years [1]. With the innovation of prostheses and the optimization of surgical techniques, total knee arthroplasty has become a reliable method for restoring patients' function and improve their quality of life [2, 3]. In recent years, the number of total knee arthroplasty (TKA) operations has gradually increased [4]. However, another trend is that the TKA-related litigations have also been steadily on the rise with the increase in the volume of TKA [5]. According to a study by McWilliams *et al*. [5], the number of claims after TKA increased by 46% between 2002 and 2010. According to the statistics, joint reconstruction surgeons are twice more likely to be sued for medical damage than other doctors, with nearly 80% of joint reconstruction surgeons having been sued at least once in their careers, and more than 50 percent of lawsuits taking place in the first 10 years of their practice [6–8].

Undoubtedly, the medical damage lawsuits not only are, up to a point, financially and psychologically traumatic to the doctors involved, and also cause great losses to the hospital. Therefore, it is necessary to understand the causes and risk factors of the litigations in order to reduce the possibility of litigation. In fact, few studies analyzed the lawsuits after knee arthroplasty. China has a different legal framework, law system and social culture from other countries, and, as a result, the causes, applicable laws and outcomes of lawsuits are different, rendering the findings of studies in other countries not directly applicable to the cases in China. What is more, no studies examined the lawsuits after knee arthroplasty in China, which makes the analysis of lawsuits after knee arthroplasty in China more helpful for Chinese surgeons. In addition, unlike other studies, our study documented the percentage of liabilities of the surgeons who lost their cases and analyzed the percentage of the liabilities on the part of surgeons in different cases.

This paper analyzed the medical damage cases after knee arthroplasty in the past ten years on the basis of a Chinese database and summarizes the focus of disputes to offer guidance to surgeons and help them to reduce the risk of exposure to lawsuits.

## Methods

This study is a retrospective analysis of cases from the Wolters Kluwer Legal Information Database (Wolters Kluwer China Law & Reference, The Kingdom of the Netherlands). The database contains cases officially announced by the Supreme People's Court since the founding of the People's Republic of China in 1949. It covers the entire trial processes of all civil, criminal and administrative cases. The types of adjudication documents include judgments, written orders, conciliation statements and other documents. With more than 15 million documents, it is a recognized legal information base in Chinese legal communities [9].

The keywords "knee arthroplasty" and "medical treatment" were adopted in the search of the database. All medical damage lawsuits after the first total knee arthroplasty were included, and a total of 1877 litigation cases were found. The inclusion criteria for each case were as follows: (1) the patient initiated a lawsuit for total knee arthroplasty; (2) the judgment was issued between June 2011 and June 2021; and (3) the doctor or hospital was the defendant; (4) The cases were repeated in the first instance, the second instance and the retrial, and only the cases in which the final judgment became effective were retained. The reasons for eliminating a case included but were not limited to: the operation the plaintiff received was knee revision surgery or unicompartment knee arthroplasty; the defendant was not a doctor or a hospital, the litigation was about a traffic accident.

Data, including the patients’ age, sex, cause of litigation, outcome of the case, amount of compensation sought by the patient and actual amount of compensation awarded, were collected.

Regarding the outcome of the case, by referring to the definition of defeat in the law, the party offering treatment is defined as having lost if the court finds that there is medical negligence during treatment and assumes the corresponding liability according to the degree of fault participation. Simply put, a surgeon loses if he or she has to pay out, and conversely, a surgeon wins if he or she does not pay out during the proceedings, and a liability range between 50% and 100% is defined as the surgeon being primarily responsible.

We entered all of the collected data into an Excel spreadsheet (Excel, Microsoft Corporation, Redmond, Washington) upon deleting the information that identified the patient. Given the impact of inflation, all compensation amounts were adjusted to the 2020 level through the Consumer Price Index (CPI). In addition, all compensation amounts were denominated in *Renminbi* (¥ RMB or CNY). Descriptive statistical analysis was performed mainly using SPSS software package (V24.0, IBM Corporation, Armonk, New York). A *P* < 0.05 indicated that the difference was statistically significant.

## Results

**Fig. 1:**
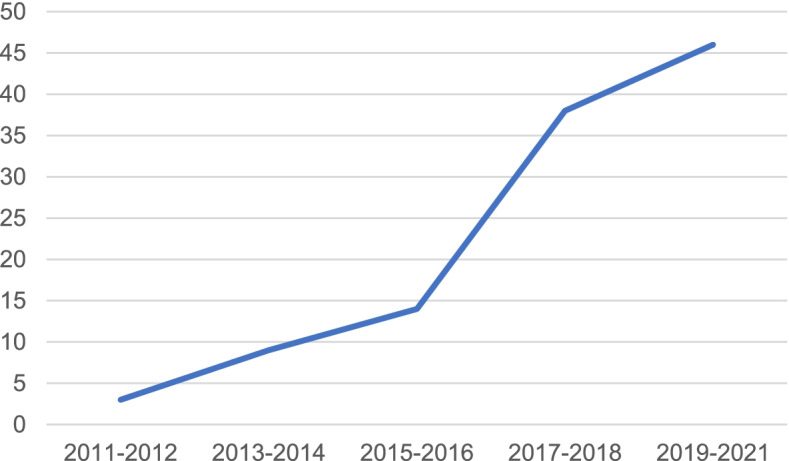
Changes in the number of lawsuits by year

**Fig. 2:**
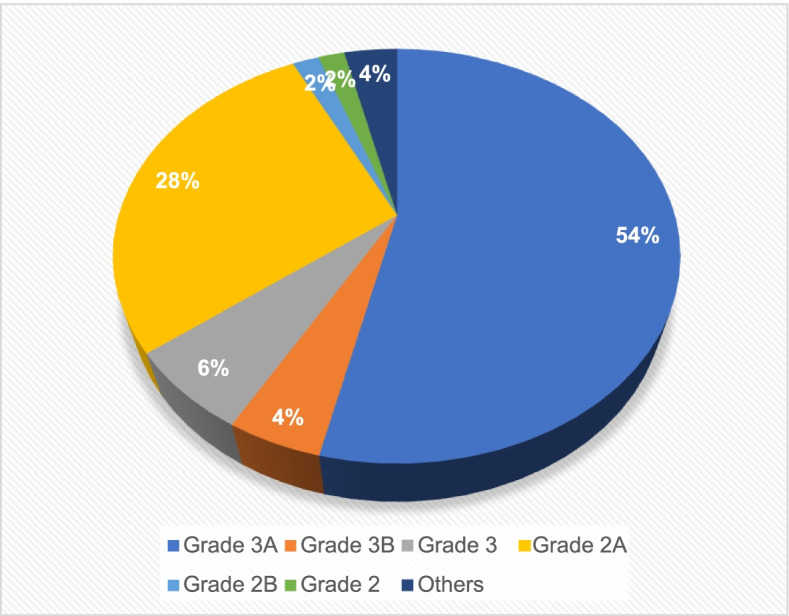
Hospital level

Finally, 110 cases of post-TKA litigation were included, and the number of litigation cases increased year by year (see Fig. 1), with 3A hospitals having more cases (see Fig. 2). In addition, the location data of the litigation cases are given in Table 1. Among the plaintiff patients, 40 were male (36.3%), and 70 were female (63.6%). The age at the time of litigation ranged from 27 to 84 years old, with an average age of 66.0 (±10.8) years. Eighty-one (73.6%) patients were under 75 years old (see Table 2).

**Table 1:** Location information of litigation cases

Province	Number
Shandong province	17
Jiangsu Province	14
Beijing	9
Anhui Province	8
Liaoning Province	8
Sichuan Province	7
Henan Province	6
Chongqing Municipality	6
Fujian Province	5
Hebei Province	4
Heilongjiang Province	3
Hubei Province	3
the Nei Monggol Autonomous Region	3
Shaanxi Province	3
Shanghai Municipality	3
Jilin Province	2
Yunnan Province	2
Gansu Province	1
the Ningxia Hui Autonomous Region	1
Qinghai Province	1
Shanxi Province	1
Tianjin Municipality	1
the Xinjiang Uygur Autonomous Region	1
Zhejiang Province	1

**Table 2:** Basic condition of the patients

Basic situation	Less than 75 years old	More than 75 years old	Total	Percent
Male	27	13	40	36.3%
Female	54	16	70	63.6%
Total	81	29	110	100%
Percent	73.6%	16.4%	100%	

In all the cases, the top three causes of litigation were infection (43.6%), postoperative dysfunction (16.4%) and persistent/waking pain (12.7%) (see Table 3). Most of the patients with infections needed revision to reconstruct the knee function (see Table 4). However, the number of post-infection reoperations ranged from 1 to 6, with an average of 2.34 (±1.45) operations. Moreover, the course of disease lasted from 3 months to 6 years and 7 months, with an average of 2.24 (±1.83) years.

**Table 3:** The number of various causes of action, the percentage of the total cases, the losing rate, the primary responsibility rate of doctors (50% ≤ doctor responsibility ≤ 100%), the average amount of compensation and the average amount of claim among all the litigation cases of total knee arthroplasty included in the study.

Cause of litigation	Number of cases	Percentage	The losing rate	The primary responsibility rate	The average amount of compensation	The average amount of claim
All cases	110	100%	90.9%	53.6%	¥213882.53(±285924.60)	¥472493.06 (±513106.45)
Infection	48	43.6%	95.8%	58.3%	¥209503.35(±228028.40)	¥464714.81(±385348.33)
Postoperative dysfunction	18	16.4%	88.9%	55.6%	¥79692.80(±69558.18)	¥233276.75(±202783.47)
Persistent / worsening pain	14	12.7%	78.6%	28.6%	¥68139.96(±70257.01)	¥207991.02(±176414.01)
Postoperative ischemic stroke	11	10.0%	81.8%	27.3%	¥392531.17(±697223.99)	¥778560.30(±1107434.12)
Death	9	8.2%	77.8%	33.3%	¥206881.60(±177197.91)	¥466866.64(±214933.66)
Prosthesis loosening	7	6.4%	100.0%	71.4%	¥153176.14(±150507.37)	¥357777.03(±248728.29)
Amputation	7	6.4%	100.0%	85.7%	¥629762.87(±540939.30)	¥928853.54(±553279.38)
Nerve injury	6	5.5%	100.0%	83.3%	¥196853.50(±97310.99)	¥ 525039.94(±430878.06)
Poor wound healing after operation	6	5.5%	83.3%	66.7%	¥93341.26(±121401.92)	¥264019.72(±371551.51)
Poor alignment	4	3.6%	100.0%	75.0%	¥203852.46(±198646.30)	¥577025.26(±577025.26)
Deep venous thrombosis	4	3.6%	100.0%	25.0%	¥199786.38(±180058.65)	¥713513.85(±345839.92)
Vascular injury	2	1.8%	100.0%	100.0%	¥345857.07(±247082.86)	¥696216.30(±634538.81)

18 patients (16.4%) had 2 complications and 9 patients (8.2%) had 3 or more complications. Some of the data are not recorded in the table because the quantity is too small.

**Table 4:** Details of infection

Details of infection	Early infection	Delayed infection	Late infection	Total
Revision	9	10	3	22
Arthrodesis	1	2	1	4
Amputation	1	2	1	4
Unoperated	7	9	2	18
Total	18	23	7	48

Early infection: Infection was found less than 3 months after operation; Delayed infection: Infection was found from 3 months to 24 months after operation; Late infection: Infection was found more than 24 months after operation.

In all the cases, 100 doctors lost the case (90.9%), 8 doctors won (7.3%), and 2 cases settled (1.8%). In the cases in which doctors lost, 9 doctors was held 100% responsible (8.2%), 41 doctors 0–50% responsible (37.3%), 36 doctors 50–100% responsible (32.7%), and in 14 cases, each party was held 50% responsible (12.7%).

The court found that the main reason for losing the case was "diagnosis and treatment errors" (67.3%) and infections caused by various errors were still on the top (45.9%). However, the rate of the doctor losing the lawsuit due to complications caused by operational errors, such as nerve injury, vascular injury, poor alignment, and amputation, was the highest, ranking the first place and the proportion of responsibility on the doctor’s part increased significantly (*P* < 0.05) (see Table 3). Other common reasons for losing the case were "neglect, failure to diagnose and treat in time" (14.5%), "incomplete or faulty in fulfilling the obligation of disclosure" (10.9%), *etc*. (see Table 5). Unexpectedly, death (77.8%) did not lead to a higher loss rate.

**Table 5:** The number and proportion of reasons determined by the court to lose the case in the collection of litigation cases of total knee arthroplasty

The court decided the reason for losing the case.	Number	Percentage
There are mistakes in the process of diagnosis and treatment.	74	67.3%
Pay no attention to it and fail to make timely diagnosis and treatment.	16	14.5%
The performance of the obligation to inform is not comprehensive or at fault.	12	10.9%
There is a defect in the case / the case is not written in time.	10	9.1%
Fault in preoperative risk assessment and risk avoidance	6	5.5%
The preoperative examination is not detailed and non-standard.	6	5.5%
Inaccurate grasp of surgical indications	5	4.5%
Insufficient postoperative rehabilitation guidance	4	3.6%
Missed diagnosis and missed treatment	3	2.7%
Tampering with and forging medical records	2	1.8%
The bar code of the prosthesis is lost.	1	0.9%
The scope of practice of economic doctors is not in line with the regulations.	1	0.9%
No preoperative measurement	1	0.9%

The amount of compensation for lost cases of doctors varied greatly, ranging from 5388.8 yuan to 2.145 million yuan, with an average compensation amount being 213882.53 (±285924.60) yuan. Complications such as amputation, postoperative ischemic stroke, vascular injury, periprosthetic fracture and infection were more likely to lead to higher compensation (see Table 3). The amount of amputation compensation was significantly higher than the average compensation (*P* < 0.05).

Moreover, several subgroup analyses were conducted, with two groups divided in terms of the amount of compensation paid, and subjects above the average amount of compensation designated as the high-compensation group and those below the average amount of compensation as the low-compensation group. For the purpose of avoiding the great error in the statistics of the litigation causes, we put the unpaid cases in the low-compensation group. There were 77 cases in the low-compensation group and 33 cases in the high one. For the low-compensation group, the major causes of action were infection, postoperative dysfunction and persistent/worsening pain, which were the same as the main causes of litigation overall. However, for the high-compensation group, the main causes of action were infection, amputation and postoperative ischemic stroke, which were closer to the complications that tend to lead to higher compensation above (see Table 6). As for the liability ratios, the high-compensation group was dominated by physicians being held primarily liable while in the low-compensation group, although the highest ratio of liability on the part of the doctors was 70%, the doctors were still considered to be secondary in the liability (see Table 7).

**Table 6:** Differences in causes of litigation between the high- and low-compensation groups

Low-compensation group			High-compensation group		
Cause of litigation	Number of cases	Percentage	Cause of litigation	Number of cases	Percentage
Infection	29	37.7%	Infection	19	57.6%
Postoperative dysfunction	16	20.8%	Amputation	5	15.2%
Persistent / worsening pain	13	16.9%	Postoperative ischemic stroke	5	15.2%
Postoperative ischemic stroke	6	7.8%	Nerve injury	4	12.1%
Poor wound healing after operation	5	6.5%	Death	4	12.1%
Death	5	6.5%	Deep venous thrombosis	2	6.1%
Prosthesis loosening	4	5.2%	Postoperative dysfunction	2	6.1%

**Table 7:** Differences in the responsibility rate between the high- and low-compensation groups

Low-compensation group			High-compensation group		
The responsibility rate	Number of cases	Percentage	The responsibility rate	Number of cases	Percentage
70%	12	15.6%	100%	7	21.2%
0%	10	13.0%	70%	6	18.2%
50%	9	11.7%	50%	5	15.2%
30%	7	9.1%	80%	4	12.1%
40%	6	7.8%	75%	2	6.1%
20%	5	6.5%	60%	2	6.1%
25%	5	6.5%	20%	2	6.1%
35%	5	6.5%	85%	1	3.0%
10%	5	6.5%	65%	1	3.0%

In the low-compensation group, the proportional responsibility of the 10 cases was 0, and the litigation causes included death, infection, dysfunction, persistent/worsening pain and postoperative ischemic stroke, with each cause of litigation involving two cases. Since there was no mistake in the process of diagnosis and treatment, the doctor’s proportional responsibility was found to be 0. All unpaid cases were cases with 0 proportional responsibility. Even so, in two cases, compensation was paid for infection and persistent/worsening pain. The reason for the compensation was that, despite absence of causal relationship between the patient’s adverse consequences and the doctor’s diagnosis and treatment, the patient should be partially compensated owing to insufficient information from the doctor.

In the high-payment group, the proportional responsibility of two cases was 20% with a high payment. One case was an case of infection, in which the reason was that the doctor neglected the infection focus (suspected abscess) of possible infection, did not control it thoroughly, and failed to fulfill the obligation of full attention, as a result, taking the secondary responsibility. However, since the patient with infection deteriorated into sepsis and finally died, the total payment amount increased. Although the doctor's payment amount accounted for only 20% of the total, it was still a huge sum of payment. In another case, the patient was at a certain risk for cerebral infarction preoperatively, but the doctor still performed the operation. The court ruled that the doctor had a certain fault in the selection of operation opportunity, risk foresight and risk avoidance, and should take a secondary responsibility. Nevertheless, a serious sequela developed, including hemiplegia and language disorder (aphasis), leading to an increase in the overall compensation and the final high compensation on the part of doctor.

## Discussion

In recent years, the litigations related to total knee arthroplasty have gradually increased with the increase in the amount of total knee arthroplasty [5]. Scott *et al*. [10] found that up to 20% of patients were not satisfied with the results after knee arthroplasty, and the dissatisfactions tend to lead to litigation.

The increase in the number of knee arthroplasty-related cases may, in part, result from the expansion of indications for the procedure. For example, if a younger patient or a patient with mild pain is treated surgically, lawsuits may be more likely. Our data showed that a higher proportion of lawsuits are filed by relatively young patients and female patients, and the result is consistent with the finding of Järvelin *et al*. [11]. The possible reason for this is that some young patients and female patients are less able to accept adverse postoperative outcomes and are prone to file lawsuits in the event of adverse outcomes that are substantially different from what was psychologically expected, and this even applies to patients with less postoperative pain. Some studies [12–14] examined the reasons why older patients do not file lawsuits and are more receptive to adverse outcomes in their later years because they feel that the financial loss to them is lower. Similarly, men are more receptive to adverse outcomes than women. Therefore, in dealing with relatively young patients, patients with less pain and female patients, doctors should exercise caution, make effort to minimize mistakes, strengthen communication, and try to lower patients' expectations in order to reduce the risk of litigation.

We found that, among all cases, the three most common causes of litigation were infection, postoperative dysfunction and persistent/waking pain, and other studies yielded similar results. Gibon *et al*. [15] showed that infection, nerve injury and unsatisfactory results were the top three causes of litigation. McWilliams *et al*. [5] exhibited that infection and surgical errors were the most common complications leading to litigation. Different from this study, a study by Patterson *et al*. [16] demonstrated that chronic pain, unsatisfactory results and nerve injury were the three most common complications leading to litigation. Although the results of different studies varied, they all revealed a similar trend, *i.e*., infection is still the most important issue in knee arthroplasty. Close attention should be paid to infection throughout the entire process of diagnosis and treatment, and necessary measures should be taken, including inquiry of medical history, preoperative examinations, infection risk assessment, perioperative use of antibiotics, aseptic procedures during the operation, clean dressing changes after the operation, and so on. In some lawsuit cases involving infection, preventive measures were constantly in place throughout the entire treatment process, *i*.*e*., the surgeons were not at fault and they eventually won the lawsuit.

It is worth noting that the common causes of failure after knee arthroplasty were not exactly the same as the common causes of litigation. Bozic *et al*. [17] showed that the three most common causes of knee arthroplasty failure in the United States were infection, mechanical loosening and implant failure/fracture. In Japan [18], mechanical loosening, infection and wear/osteolysis were the most common causes of failure. In China [19], the most common causes were infection, mechanical loosening and ankylosis of the knee joint. Only in the cases of infections were the common causes of failure after knee arthroplasty identical to the complications that led to litigation. Consequently, doctors and patients differed in their levels of attention to postoperative knee joint abnormalities. Litigation easily occurred when the results of the operation were different from those expected by patients [15]. With patients’ requirements for the quality of life increasing, their requirements for postoperative pain and functional improvement are also higher. If a surgeon fails to warn a patient of the possible postoperative pain and malfunction, the patient is more likely to file a lawsuit if his or her pain does not improve significantly or even worsens or the joint malfunctions after the operation. Consequently, when communicating with a patient before surgery, doctors should explain, in detail, not only the complications but also the risk of pain and dysfunction after surgery. In addition, it is necessary to include this information in the informed consent form before the operation to lower the expectations of patients and thereby reduce the risk of litigation. Moreover, more attention should be given to perioperative pain management and postoperative rehabilitation guidance.

The loss rate of doctors in this study was significantly higher than that in studies based in other countries [5, 11, 20]. However, the main responsibility rate was similar to that in other studies. The possible reason might be that, in the lawsuits, the patient is generally taken as a weak party in Chinese culture, and judges tend to make decisions that are more in favor of patients from a reasonable point of view in litigation. In some lawsuits, doctors only accounted for a minor responsibility and were still ruled to lose the case. In these cases, the compensation was relatively low and more acceptable to doctors, although the loss rate and main responsibility rate of Chinese doctors were higher than those in other countries. What is more, the settlement rate in this paper was low, which might be ascribed to the fact that it is usually difficult for doctors and patients to reach a consensus concerning the amount of compensation. From these data, the amount of compensation claimed by patients was significantly higher than the actual amount of compensation (*P* < 0.05). As a result, the number of settled cases was low. The amount of compensation in all the cases varied greatly, ranging from 5388.8 yuan to 2.145 million yuan, with an average compensation amount of 213882.53 (±285924.60) yuan. Among all the complications, the complications that led to more severe disability or a longer course of disease, such as amputation and infection, tended to receive higher compensation. This is related to the content of personal injury compensation in China, which stipulates that if a patient is found to be disabled and to have sustained higher level of disability, compensation to be paid in a lump sum is also higher, while for complications of protracted duration, the longer the period of care and the higher the level of care determined, the higher the compensation. In this study, although the doctors' proportional responsibility made up only 20% in some cases, the total amount of compensation was huge due to the severity of complications or even death of patients, resulting in the high amount of compensation. Therefore, it is necessary to pay full attention to the complications that may cause more severe disabilities or prolonged course of disease, to avoid high compensation paid by doctors.

The main reason for losing cases was "diagnosis and treatment error" (67.3%). Doctors lost lawsuits and the proportion of responsibility increased significantly due to the complications caused by mishandling during surgery, such as nerve injury, vascular injury, poor alignment, amputation, *etc.* (*P* < 0.05). This was believed to be the errors principally on the part of doctors, which is coincident with the results of Novi *et al*. [21]. Although these complications cannot be completely eliminated, improving operative skills and reasonable training can reduce the occurrence of complications [22, 23].

Other reasons for losing the lawsuit, including "not paying attention to, not giving timely diagnosis and treatment" and "the performance of the obligation of disclosure is not comprehensive or faulty", also have a certain reference value. Regardless of the complications, doctors were generally held responsible for delayed diagnosis and treatment of complications [15].

In the cases included in this study, the one with the highest payout is worth mentioning. After the patient showed signs of postoperative ischemic stroke, the patient's family members repeatedly communicated with the surgeon and requested further treatment, but the surgeon did not take it seriously and kept on observing the patient instead of holding a consultation in time, resulting in delayed treatment and paralysis of the patient's extremities. Eventually, the patient was found to have Grade 1 disability and was awarded a large sum of compensation. In this case, if the doctor had held a consultation and managed the case initially, not only might the lawsuit have been avoided but also the patient might have been saved in time from any serious adverse consequences. Therefore, attention should be given to the issue of complications. It is necessary to respond proactively and engage in communication with patients and their family members. Early diagnosis and treatment can not only minimize the harm of complications but also reduce the possibility of litigation [24]. Additionally, although a higher number of patients have filed lawsuits for persistent/worsening pain, it is difficult for the court to uphold subjective descriptive damages raised by the patients, whereas the existence of substantial damages to the patient due to the fault of the doctor is more likely to be sustained.

Informed consent and doctors’ communication with their patients in the doctor-patient relation are now playing increasingly more important roles with the elevation of people's awareness of their legal rights, including self-protection. Full communication allows patients to obtain sufficient information during the medical process. Medical activities with patients' knowledge and consent can significantly reduce the risk of medical damage litigation, which has been confirmed by multiple studies [25–28]. Patients should be informed of the potential risks, damage, benefits, success probability and alternatives of the treatment regimen before treatment [29], including the risks associated with litigation. In some cases where the doctor's proportional responsibility was 0, there were still cases of payment due to insufficient notification. Even if the patient's damage bore no correlation with the doctor's diagnosis and treatment, it might also result from inadequate notification. Therefore, great attention should be paid to the doctor-patient communication throughout the entire process of diagnosis and treatment. However, it is also important to note that communication also requires tact, and a balance should be struck between informing patients and scaring them. In addition, the communication content should be recorded in detail in each case, and studies have shown that informed consent often covers only a small number of items [30, 31], which is an area that we should improve.

It is necessary to be vigilant against other reasons for losing cases, such as "defective cases/cases not written in time", "faults in preoperative risk assessment and risk avoidance" and "incomplete preoperative examination, non-standard". We should minimize these kinds of situations and reduce the possibility of losing lawsuits due to errors during the process of diagnosis and treatment.

It is worth noting that litigation of medico-legal issue is a highly sophisticated process. Judges, prosecutors, medical professionals, lawyers, and even patients play important roles in the process. So, all the players need education and disciplinary training to ensure that the whole process is reasonable and fair.

The limitations of this study are as follows: (1) Due to the lack of a universal compulsory litigation database in China, it is impossible for us to know the true number of lawsuits after total knee arthroplasty. Although this study included 110 TKA-related litigation cases that met our criteria, these cases might only represent a portion of the medical damage litigations during this period of time. It is possible that some cases were not uploaded to Wolters Kluwer’s Advanced Legal database, but all cases were not screened during the process of uploading. Therefore, it will not lead to bias in the results. (2) Our study included only patients with primary total knee arthroplasty, excluding knee revision and unicompartmental knee arthroplasty.

## Conclusions

The risk of litigation caused by infection is huge, and operational errors are more likely to cause lost cases than any other factors during the process of diagnosis and treatment. Therefore, the prevention of infection and the avoidance of operational errors are very important. The risks of litigation can be reduced by fully evaluating the risk and improving the surgical skills. However, for pain and dysfunction, the outcome of the operation remains the decisive factor. Full and reasonable communication and lowering the psychological expectations of patients are reliable approaches to avoiding litigation. Furthermore, avoiding the complications of disability or a long course of disease can significantly reduce the amount of compensation. Paying full attention to the reactions of patients and their families, a timely diagnosis, treatment and meticulous recording of medical records can also effectively reduce the risk of litigation and loss.

## Data Availability

The datasets used and/or analyzed during the current study are available from the corresponding author on reasonable request.

## Abbreviations

TKA: Total knee arthroplasty
CPI: Consumer Price Index
RMB: Renminbi
